# Sesamin Ameliorates Advanced Glycation End Products-Induced Pancreatic β-Cell Dysfunction and Apoptosis

**DOI:** 10.3390/nu7064689

**Published:** 2015-06-09

**Authors:** Xiang Kong, Guo-Dong Wang, Ming-Zhe Ma, Ru-Yuan Deng, Li-Qun Guo, Jun-Xiu Zhang, Jie-Ren Yang, Qing Su

**Affiliations:** 1 Department of Endocrinology, Xinhua Hospital, Shanghai Jiaotong University School of Medicine, Shanghai 200092, China; E-Mails: wnmcyaolikx@sina.com (X.K.); luckyruyuan@163.com (R.-Y.D.); 2 Department of Pharmacology, Wannan Medical College, Wuhu 241002, China; E-Mails: wy_glq@163.com (L.-Q.G.); junxiuzhang@qq.com (J.-X.Z.); 3 Department of Pharmacy, Wannan Medical College, Wuhu 241002, China; E-Mail: guodong201@163.com; 4 Department of General Surgery, Xinhua Hospital, Shanghai Jiaotong University School of Medicine, Shanghai 200092, China; E-Mail: mmz666@163.com

**Keywords:** sesamin, advanced glycation end products, MIN6 cell, reactive oxygen species, apoptosis

## Abstract

Advanced glycation end products (AGEs), the direct modulators of β-cells, have been shown to cause insulin-producing β-cell dysfunction and apoptosis through increase of intracellular reactive oxygen species (ROS) production. Sesamin has been demonstrated to possess antioxidative activity. This study was designed to investigate whether sesamin protects against AGEs-evoked β-cell damage via its antioxidant property. The effects of sesamin were examined in C57BL/6J mice and MIN6 cell line. In *in vivo* studies, mice were intraperitoneally injected with AGEs (120 mg/kg) and orally treated with sesamin (160 mg/kg) for four weeks. Intraperitoneal glucose tolerance and insulin releasing tests were performed. Insulin content, ROS generation and β-cell apoptosis in pancreatic islets were also measured. In *in vitro* studies, MIN6 cells were pretreated with sesamin (50 or 100 μM) and then exposed to AGEs (200 mg/L) for 24 h. Insulin secretion, β-cell death, ROS production as well as expression and activity of NADPH oxidase were determined. Sesamin treatment obviously ameliorated AGE-induced β-cell dysfunction and apoptosis both *in vivo* and *in vitro*. These effects were associated with decreased ROS production, down-regulated expression of p67^phox^ and p22^phox^, and reduced NADPH oxidase activity. These results suggest that sesamin protects β-cells from damage caused by AGEs through suppressing NADPH oxidase-mediated oxidative stress.

## 1. Introduction

Diabetes mellitus (DM) is characterized by hyperglycemia and is becoming a public health problem of considerable magnitude. Advanced glycation end products (AGEs) are generated from nonenzymatic glycoxidation of proteins and lipids, and the formation of AGEs is greatly accelerated by prolonged hyperglycaemia in patients with DM [1,2,3]. Increasing evidence indicates that the accumulation of AGEs play a critical role in the pathogenesis of diabetic complications [4,5]. Interestingly, recent studies have pointed out that AGEs directly cause insulin-producing β-cell dysfunction and apoptosis *in vivo*, and contribute to the development of DM [6,7,8]. Furthermore, the AGE-induced β-cell damage has been demonstrated to be associated with the increase in intracellular reactive oxygen species (ROS) production in our previous studies [9,10] and other reports [7,11,12].

Sesamin is a natural lignan found in sesame oil and seeds, and exerts a variety of pharmacological properties. Both clinical and experimental studies have illustrated the lipid-lowering, antioxidative and antihypertensive effects of sesamin [13,14,15,16,17,18]. A recent study has shown that sesamin ameliorates the insulin resistance in KK-Ay mice, a kind of type 2 DM model [19]. However, to the best of our knowledge, no studies have been conducted on the effects of sesamin on AGE-induced β-cell lesion. Accordingly, the current study was designed to investigate whether sesamin could protect against AGE-induced β-cell dysfunction and apoptosis both *in vitro* and *in vivo*, and to further clarify whether the protective effects of sesamin were attributed to its antioxidant properties.

## 2. Materials and Methods

### 2.1. Drugs and Reagents

All of the reagents were purchased from Sigma unless stated otherwise. Sesamin (>94% purity) was provided by Tianyi Lvbao Technology Co. (Wuhu, China). Its structure and invention patent number were described in our previous study [13]. Mouse insulin ELISA Kit was purchased from Shibayagi Co. (Shibukawa, Japan). Terminal deoxynucleotidyl transferase dUTP nick end labeling (TUNEL) and Cell Death Detection ELISA^Plus^ assay kits were purchased from Roche CO. (Indianapolis, USA). 2’,7’-dichlorodihydrofluorescin diacetate (DCFH-DA) and dihydroethidium (DHE) probes were purchased from Beyotime Biotechnology Inc. (Nantong, China). Polyclonal rabbit anti-insulin antibody was purchased from Abcam Inc. (Cat No.: ab63820, Cambridge, UK). Polyclonal rabbit anti-nicotinamide adenine dinucleotide phosphate (NADPH) oxidase subunits p22^phox^ (Cat No.: sc-20781) and p67^phox^ (Cat No.: sc-15342) antibodies were purchased from Santa Cruz Biotechnology Inc. (Santa Cruz, CA, USA). Polyclonal rabbit anti-cleaved caspase 3 (Cat No.: AC033) and poly (ADP-ribose) polymerase (PARP, Cat No.: AP102) antibodies as well as monoclonal mouse anti-tubulin antibody (Cat No.: AT819) were purchased from Beyotime Biotechnology Inc. (Nantong, China).

### 2.2. Preparation and Validation of AGEs

AGEs were prepared as described by Makita *et al.* [20]. In brief, 50 mg/mL of bovine serum albumin (BSA) were incubated under sterile condition with 0.1 M of glyceraldehyde in 0.2 M of phosphate buffer (pH 7.4) for seven days. The unincorporated sugar was removed by dialysis. Non-glycated BSA was incubated under the same condition except for the absence of glyceraldehyde as a negative control. The content of AGEs was determined using the standard spectrum λ_ex_ 370 nm/λ_em_ 450 nm with a fluorence microplate reader. The AGE preparation was tested for endotoxin using a limulus amebocyte lysate reagent (Associates of Cape Cod, Inc., East Falmouth, MA, USA) and was confirmed to have an endotoxin level of less than 15 EU/L. Furthermore, the prepared AGEs and BSA were assessed for changes in molecules by SDS-polyacrylamide gel electrophoresis (SDS-PAGE) using a 10% gel and Coomassie Blue staining.

### 2.3. Laboratory Rodent Studies

Ten-week-old male C57BL/6J mice were obtained from SLAC Laboratory Animal Co. (Shanghai, China). All of the mice were maintained in specific pathogen-free facilities at the experimental animal center of Xinhua Hospital. All studies were approved by the Institutional Animal Care and Use Committee (IACUC) at the Xinhua Hospital, Shanghai Jiaotong University School of Medicine. All mice received humane care in accordance with the Guide for the Care and Use of Laboratory Animals published by the US National Institutes of Health (NIH publication no. 85-23, revised 1996).

Mice were separated into the following groups (*n* = 6 each group): BSA control group, AGE model group and sesamin treatment group (AGEs plus sesamin, given sesamin by gavage at the daily dose of 160 mg/kg body weight). BSA or AGEs were administered intraperitoneally daily for 4 weeks with the dosage (120 mg/kg body weight) according to a previous report [6]. Based on previous studies, the dosage of sesamin has the sufficient *in vivo* antioxidant capacity [13,17]. During the entire period of the experiment, body weight (BW) and food consumption were measured weekly.

### 2.4. Intraperitoneal Glucose Tolerance Test (GTT), Insulin Releasing Test (IRT) and Intraperitoneal Insulin Tolerance Test (ITT)

An intraperitoneal GTT was used to assess glucose tolerance. After fasting overnight, mice were intraperitoneally injected with 10% glucose solution (1.5 mg/g body weight), and glucose levels were determined at 0, 30, 60, 90, and 120 min by a glucometer. For measuring glucose stimulated insulin secretion, blood was collected at 0, 30, and 60 min after glucose loading, and insulin levels were determined using the ELISA Kit.

After 6 h fasting, ITT was performed in mice. Blood samples were collected from tails before and 15, 30, 45, and 60 min after an intraperitoneal injection of human regular insulin (0.75 U/kg). Glucose levels were measured using a glucometer.

### 2.5. Immunofluorescent Staining for Insulin in Mice Pancreatic Islets

A part of the pancreas was fixed in 4% paraformaldehyde, embedded in paraffin, and cut into 5-μm sections. For immunofluorescent staining, fixed pancreatic sections were heated for 15 min in boiling 10 mM citrate buffer for antigen retrieval. Sections were subsequently probed with anti-insulin antibody (1:200), followed by incubation with specific secondary antibodies. Nuclear staining was achieved by incubating with DAPI. Sections were photographed by fluorescent microscopy and analyzed using Image J software as described in our previous report [21]. Briefly, mean fluorescence intensity (MFI) of insulin staining, reflecting the insulin content, was quantified in ten randomly selected pancreatic islets of each mouse. Data were pooled to calculate a mean value, and a statistical analysis was applied to compare the results obtained from different experimental groups.

### 2.6. Assessment of Apoptosis in Mice Pancreatic Islets

The terminal deoxynucleotidyl transferase dUTP nick end labeling (TUNEL) assay kit was used to evaluate the islet apoptosis. Briefly, 5 μm sections were incubated with TUNEL reagent for 1 h in dark. After rinsing with PBS, the 3,3’-diaminobenzidine reagent was added. In addition, the positive control includes permeabilization of sections with deoxyribonuclease 1 to induce DNA strand breaks. The slides were mounted and photographed under light microscope. The TUNEL positive cells and total islet cells were counted manually in ten randomly selected islets of each mouse under a 20× objective microscope by an investigator who was blinded to the experiments. The results were adjusted for β cell area in serial sections stained with insulin and used to calculate the TUNEL positive β cells per islets.

### 2.7. In Situ Detection of ROS Production in Mice Pancreatic Islets

A part of the pancreas was included in O.C.T. embedding medium. Sections of 10 μm were obtained in a freezing microtome. Sections were incubated for 30 min with 10 μM DHE to evaluate pancreatic superoxide anion levels *in situ*. DHE is oxidized by superoxide anion to yield ethidium, which stains DNA. Sections were photographed and MFI of DHE was quantified in ten randomly selected pancreatic islets of each mouse with Image J software. Data were pooled to calculate a mean value and the results were statistically evaluated.

### 2.8. Cell Culture and Treatment

The pancreatic MIN6 β-cell line was a gift from the Institute of Endocrinology of Rui Jin Hospital affiliated with the Shanghai Jiao Tong University School of Medicine. As described in our previous study [9], MIN6 cells (15–30 passages) were grown in Dulbecco’s modified Eagle’s medium (DMEM) containing 10% FBS, 50 μM 2-mercaptoethanol, 100 U/mL penicillin, and 100 μg/mL streptomycin. MIN6 cells were grown to confluence, pretreated with sesamin (50 or 100 μM) for 2 h, and then stimulated with AGEs (200 mg/L) for 24 h.

### 2.9. Measurement of Insulin Secretion in MIN6 Cells

After treatment, the MIN6 cells were starved in Krebs buffer containing 0.2% bovine BSA with 3 mM glucose for 30 min. The cells were then incubated in Krebs buffer with 3 or 25 mM glucose for 60 min. An aliquot of the buffer was taken and insulin release was measured by ELISA.

### 2.10. Western Blot Analysis

As described in our previous study [22], equal amounts of proteins were applied to a 10% or 12% SDS-polyacrylamide separating gel and transferred to a polyvinylidene difluoride (PVDF) membrane (Millipore). After blocking with 5% skim milk or 1% BSA in Tris buffered saline with Tween 20 (TBST) at room temperature for one hour, the membrane was incubated with the primary antibodies against cleaved caspase 3 (1:500), PARP (1:800), NADPH oxidase subunit p22^ph^^ox^ (1:400), or p67^ph^^ox^ (1:250) overnight. After washing the membranes three times, the immunoblots were incubated with the appropriated secondary antibodies for 2 h. Antibody-bound proteins were detected by Millipore enhanced chemiluminescence kit. The blots were evaluated by densitometry using Image J software. The intensity of the bands was normalized to that of tubulin.

### 2.11. Detection of Cell Death

Histone/DNA complexes released from the nucleus to the cytosol (DNA fragmentation), a marker for apoptosis, were measured according to the manufacturer’s protocol.

### 2.12. Evaluation of ROS Production in MIN6 Cells

2’,7’-dichlorodihydrofluorescin diacetate (DCFH-DA) was used to evaluate intracellular ROS in the same treated MIN6 cells. DCFH-DA, a cell-permeant probe, is oxidized to form highly fluorescent DCF in the presence of hydrogen peroxide (H_2_O_2_) or low molecular weight peroxides produced by the cells. After treatment, the MIN6 cells were incubated with 10 μM DCFH-DA for 30 min at 37 °C. After incubation, the cells were washed with PBS twice, trypsinized, resuspended and immediately submitted to flow cytometry analysis.

### 2.13. Measurement of NADPH Oxidase Activity in MIN6 Cells

NADPH oxidase activity was measured as previously described [9,10]. After treatment, MIN6 cells were trypsinized, pelleted by centrifugation and re-suspended with cold Krebs-HEPES buffer. Cellular suspensions of 300 μL were put into a 96-well white plate in a luminescence reader and a dark-adapted 10 μM of lucigenin was added to start the reaction. Chemiluminescence was recorded every 15 s for 10 min. NADPH of 100 μM was added after measuring the background lucigenin chemiluminescence, with further measurement performed for another 10 min. The differences between the values obtained before and after adding NADPH were calculated, and the result represented the activity of NADPH oxidase.

### 2.14. Statistical Analysis

Values are expressed as mean ± SD. Comparisons among groups were determined by the use of one-way analysis of variance (ANOVA) followed by a Newman-Keuls test. A value of *p* < 0.05 was considered statistically significant.

## 3. Results

### 3.1. Characterization of the AGEs

**Figure 1:**
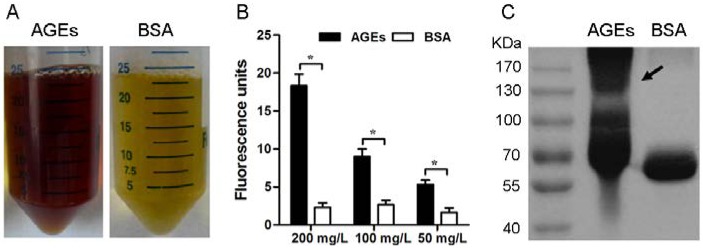
Characterization of the AGEs prepared in the current study. (**A**) Representative photos of AGEs and BSA. (**B**) Histogram represents the obvious increase in fluorescence intensity emitted from AGEs. Results are expressed as mean ± SD, *n* = 3, * *p* < 0.05. (**C**) Representative SDS-PAGE graphs of AGEs and BSA.

The formation of AGEs is characterized by fluorescence, brown color, and cross-linking [23]. Our results were consistent with the previous report [20], AGEs prepared in the current study showed the brown color (Figure 1A) and the marked increase in fluorescence intensity (Figure 1B). Additionally, AGEs appeared as a smear of large moleculars on the SDS-PAGE, whereas BSA showed tighter bands (Figure 1C). These data suggest that highly modified and polymerized albumin molecules are present in the AGE preparations.

### 3.2. Insulin Content, GTT, IRT, ITT, BW and Food Intake in Mice

**Figure 2:**
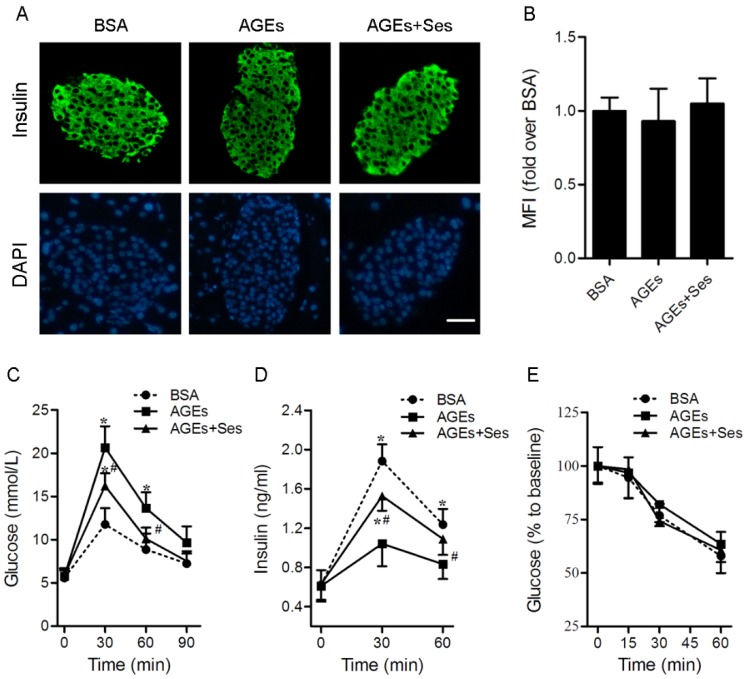
Effects of sesamin on insulin content, GTT, IRT and ITT in mice. C57BL/6J mice were injected intraperitoneally with BSA (120 mg/kg, as a control), AGEs (120 mg/kg) or AGEs plus sesamin (160 mg/kg) for 4 weeks. (**A**) Representative photomicrographs of immunofluorescent staining for insulin in pancreatic islets. Scale bar = 50 μm. (**B**) Histogram represents quantitative analysis of insulin content in each experimental group. GTT (**C**) and IRT (**D**) were performed after intraperitoneal injection of glucose (1.5 mg/g). Values are expressed as mean ± SD, *n* = 6 per group. * *p* < 0.05 *versus* BSA group. ^#^ *p* < 0.05 *versus* AGEs group. MFI: Mean fluorescence intensity; GTT: Glucose tolerance test; IRT: Insulin releasing test.

As shown in Figure 2A,B, there were no significant differences in insulin content among all experimental groups. Compared with BSA control-injected mice, impaired glucose tolerance (11.8 ± 1.9 *vs*. 20.7 ± 2.4 mmoL/L at the 30-min point, 8.9 ± 1.9 *versus* 13.6 ± 1.8 mmoL/L at the 60-min point, *p* < 0.05, Figure 2C) and glucose-stimulated insulin secretion (1.88 ± 0.17 *versus* 1.04 ± 0.23 ng/mL at the 30-min point, 1.24 ± 0.16 *versus* 0.83 ± 0.15 mmoL/L at the 60-min point, *p* < 0.05, Figure 2D) were present in AGE-treated mice, although there were no obvious changes in fasting glucose, insulin and ITT (Figure 2E). This AGE-dependent high level of blood glucose (16.2 ± 1.5 *versus* 20.7 ± 2.4 mmoL/L at the 30-min point, 10.1 ± 1.3 *versus* 13.6 ± 1.8 mmoL/L at the 60-min point, *p* < 0.05, Figure 2C) and insulin secretory defect (1.53 ± 0.15 *versus* 1.04 ± 0.23 ng/mL at the 30-min point, 1.09 ± 0.16 *versus* 0.83 ± 0.15 mmoL/L at the 60-min point, *p* < 0.05, Figure 2D) during the glucose challenge was partially improved by treatment with sesamin. Food consumption (3.17 ± 0.28, 3.11 ± 0.33 and 3.23 ± 0.37 g/day) and body weight (26.1 ± 0.93, 26.4 ± 1.2 and 26.6 ± 1.4 g) did not differ significantly among all experimental groups.

### 3.3. Apoptosis in Mice Pancreatic Islets

**Figure 3:**
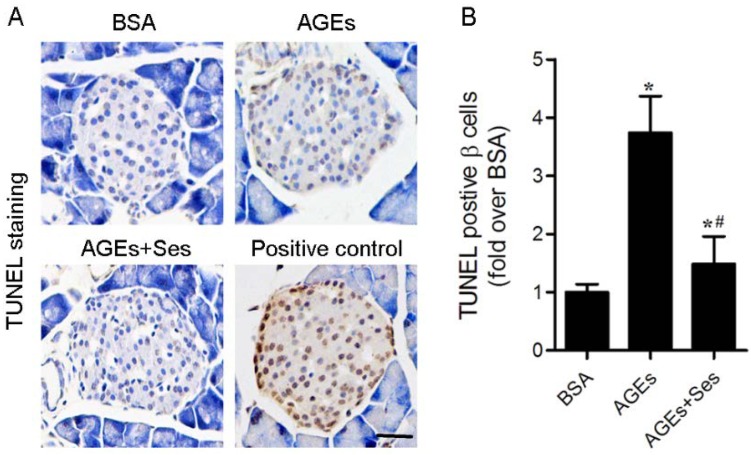
Effects of sesamin on apoptosis in mice pancreatic islets. (**A**) Representative photomicrographs of TUNEL-positive cells in mice pancreatic sections. The positive control includes permeabilization of sections with deoxyribonuclease 1 to induce DNA strand breaks. Scale bar = 50 μm. (**B**) Histogram represents quantitative analysis of TUNEL-positive β cells per islets in each experimental group. Values are expressed as mean ± SD, *n* = 6 per group. * *p* < 0.05 *versus* BSA group. ^#^ *p* < 0.05 *versus* AGEs group.

Exposure to AGEs caused pancreatic β-cell apoptosis as assessed by TUNEL staining in C57BL/6J mice (*p* < 0.05 *versus* BSA group), which was not observed in BSA-injected mice and was obviously prevented by sesamin treatment (*p* < 0.05 *versus* AGEs group, Figure 3).

### 3.4. ROS Production in Mice Pancreatic Islets

**Figure 4:**
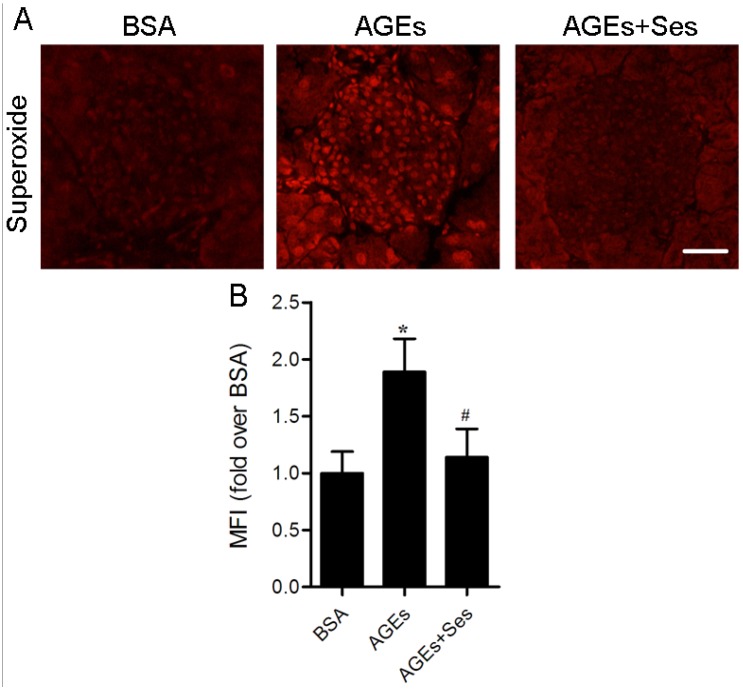
Effects of sesamin on ROS (superoxide anion) production in mice pancreatic islets. (**A**) Representative photomicrographs of mice pancreatic sections stained with DHE. Scale bar = 50 μm. (**B**) Histogram represents quantitative analysis of superoxide anion generation in each experimental group. Values are expressed as mean ± SD, *n* = 6 per group. * *p* < 0.05 *versus* BSA group. ^#^ *p* < 0.05 *versus* AGEs group. MFI: Mean fluorescence intensity.

Ethidium red fluorescence in sections of pancreatic tissues obtained from incubation with DHE was used to characterize and localize ROS (superoxide anion) production. As shown in Figure 4, the MFI of superoxide anion in AGE-treated mice islets (21.1 ± 3.2) was markedly higher than that in BSA-treated mice (11.2 ± 2.2, *p* < 0.05). Treatment with sesamin induced a significant decrease of superoxide anion production in islets (12.7 ± 2.8, *p* < 0.05 *versus* AGEs group).

### 3.5. Insulin Secretion in MIN6 Cells

**Figure 5:**
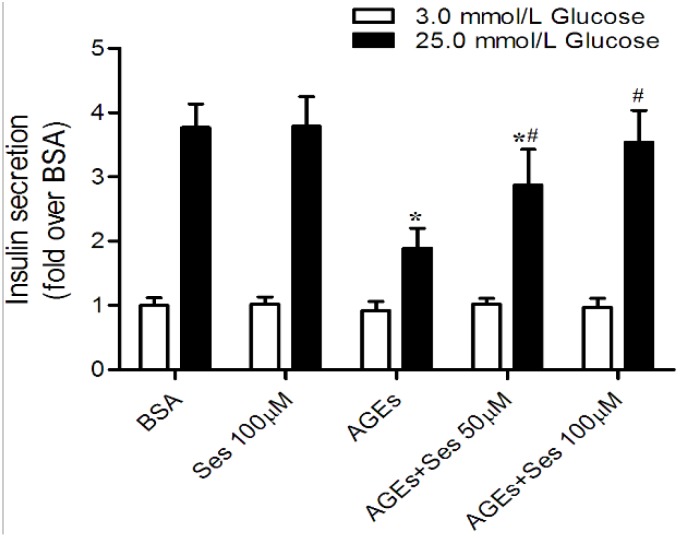
Effects of sesamin on insulin secretion in MIN6 cells. Cells were exposed to 200 mg/L AGEs for 24 h in the presence or absence of sesamin (50 or 100 μM). The cells were pretreated with sesamin 2 h prior to AGEs stimulation. MIN6 cells were also incubuted with BSA (200 mg/L) as a control. Values are expressed as mean ± SD, *n* = 4. * *p* < 0.05 *versus* BSA. ^#^ *p* < 0.05 *versus* AGEs.

To evaluate the direct effects of sesamin on AGEs-induced pancreatic β-cell dysfunction, we treated the MIN6 cells with BSA, AGEs or AGEs plus sesamin, and then conducted glucose-stimulated insulin release assays. No significant differences in insulin release were observed in MIN6 cells after being incubated with the low concentration of glucose, whereas AGE-treated MIN6 cells (14.4 ± 2.4 ng insulin/mg protein/h) secreted less insulin than BSA control (28.7 ± 2.8 ng insulin/mg protein/h, *p* < 0.05) in response to 25 mM glucose. The blunted insulin secretion in AGE-stimulated MIN6 cells was obviously rescued by treatment with 50 (21.9 ± 4.2) or 100 μM sesamin (27.1 ± 3.7 ng insulin/mg protein/h, *p* < 0.05). However, sesamin alone did not affect insulin secretion (Figure 5).

### 3.6. Apoptosis in MIN6 Cells

**Figure 6:**
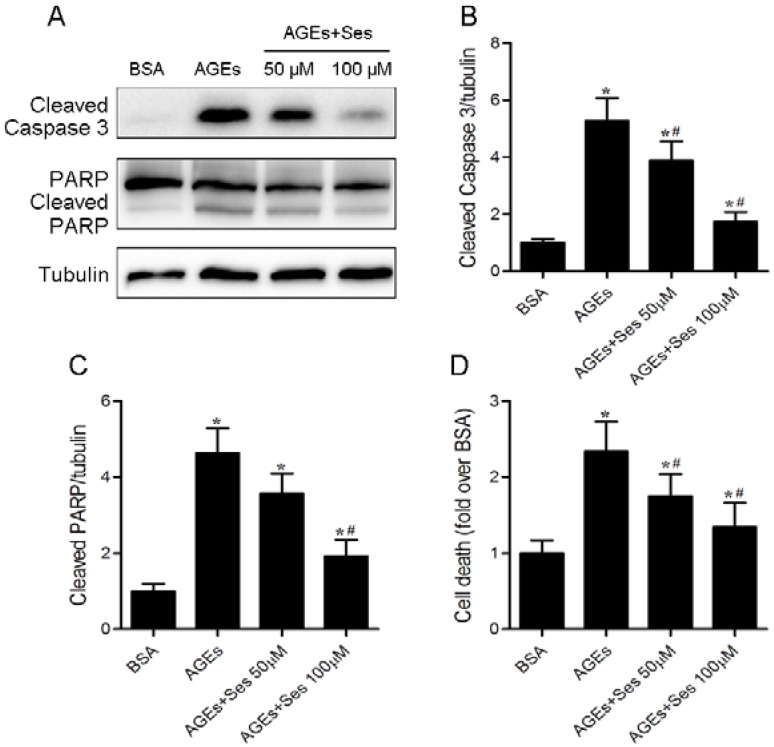
Effects of sesamin on apoptosis in MIN6 cells. Cells were prepared as in Figure 5. (**A**) Panel shows representative bands of cleaved caspase 3 and PARP. Histograms represent optical density values of cleaved caspase 3 (**B**) and PARP (**C**) normalized to the corresponding tubulin. (**D**) Apoptosis in MIN6 cells was determined by DNA fragmentation using the Cell Death Detection ELISA^Plus^ assay kit. Values are expressed as mean ± SD, *n* = 3–4. * *p* < 0.05 *versus* BSA. ^#^ *p* < 0.05 *versus* AGEs.

Caspase 3 and PARP are important mediators of apoptosis. Western blot analysis demonstrated that the expression of cleaved caspase 3 and poly (ADP-ribose) polymerase (PARP) was enhanced in AGE-incubated MIN6 cells, and these changes were decreased by sesamin treatment (Figure 6A–C). As a marker for apoptosis, the histone/DNA complexes released from the nucleus to the cytosol (DNA fragmentation) were measured. AGE-stimulated MIN6 cells had a significant increase in cell death (0.47 ± 0.08 *versus* 1.11 ± 0.17 A.U., *p* < 0.05), which was partially reversed by sesamin therapy (0.63 ± 0.14 A.U., *p* < 0.05, Figure 6D).

### 3.7. ROS Production in MIN6 Cells

**Figure 7:**
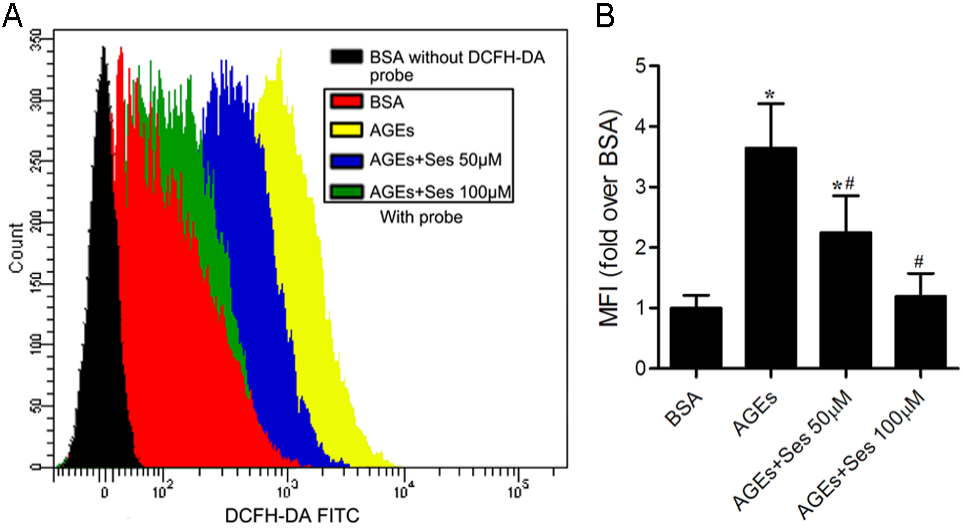
Effects of sesamin on ROS (peroxides) production in MIN6 cells. Cells were prepared as in Figure 5. (**A**) Representative pictures of ROS generation measured by flow cytometry using the DCFH-DA probe. (**B**) Histogram represents quantitative analysis of ROS accumulation in MIN6 cells. Values are expressed as mean ± SD, *n* = 4. * *p* < 0.05 *versus* BSA. ^#^ *p* < 0.05 *versus* AGEs. MFI: Mean fluorescence intensity.

We further investigated the generation of ROS (peroxides), a potential factor related to AGEs-induced pancreatic β-cell damage, by using DCFH-DA as a fluorescence probe. Flow cytometry analysis displayed that the ROS production was markedly enhanced in AGEs-incubated MIN6 cells (*p* < 0.05 *versus* BSA control). Treatment with sesamin obviously decreased the level of ROS generation (*p* < 0.05 *versus* AGEs, Figure 7). The MFI in each group was as follows: 196.8 ± 41.4, 719.6 ± 140.7, 442.2 ± 119.8, 248.6 ± 84.7.

### 3.8. Expression and Activity of NADPH Oxidase in MIN6 Cells

**Figure 8:**
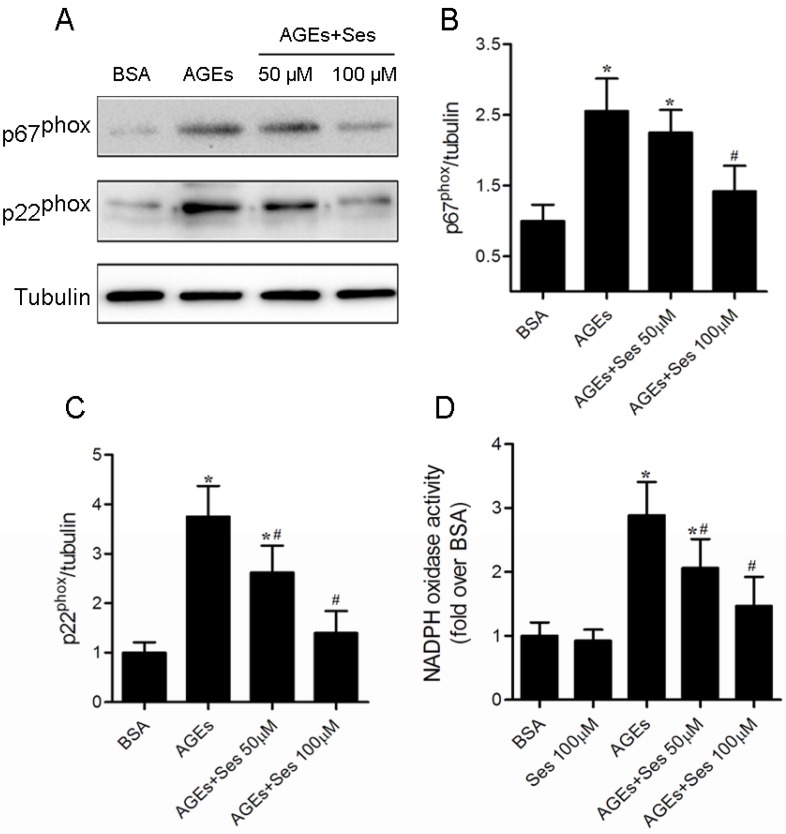
Effects of sesamin on expression of p67^phox^ and p22^phox^ as well as activity of NADPH oxidase in MIN6 cells. Cells were prepared as in Figure 5. (**A**) Panel shows representative bands of NADPH oxidase subunits p67^phox^ and p22^phox^. Histograms represent optical density values of p67^phox^ (**B**) and p22^phox^ (**C**) normalized to the corresponding tubulin. (**D**) NADPH oxidase activity in MIN6 cells was measured with the chemiluminescence method. Values are expressed as mean ± SD, *n* = 3–5. * *p* < 0.05 *versus* BSA. ^#^ *p* < 0.05 *versus* AGEs.

As mentioned in our previous studies, NADPH oxidase is the major source of ROS in β-cell incubated with AGEs [9,10]. We therefore investigated the effects of sesamin on NADPH oxidase expression and activity. The significant increase in expression of p67^phox^ and p22^phox^ and activity of NADPH oxidase were exhibited in AGE-stimulated MIN6 cells (*p* < 0.05 *versus* BSA), whereas treatment with sesamin reversed these abnormal changes (*p* < 0.05 *versus* AGEs). Treatment with sesamin alone did not affect NADPH oxidase activity (Figure 8). The NADPH oxidase activity in each group was as follows: 188.6 ± 39.7, 174.2 ± 32.7, 544.8 ± 98.1, 387.5 ± 85.7, 253.1 ± 71.0.

## 4. Discussion

The loss of pancreatic β-cell function is a central feature of both type 1 and type 2 DM. AGEs are generated and accumulated by chronically elevated blood glucose in DM, and contribute to the deterioration of DM. Consistent with previous reports [6,9], exposure to AGEs directly caused pancreatic β-cell dysfunction and apoptosis in C57BL/6J mice and MIN6 cells. These effects were not owing to the development of insulin resistance because intraperitoneal injections of AGEs did not affect fasting glucose and insulin levels and peripheral insulin sensitivity in C57BL/6J mice. Furthermore, sesamin treatment improved the AGE-induced β-cell damage *in vitro* and *in vivo*.

Enhanced levels of reactive oxygen species (ROS), such as superoxide anion and H_2_O_2_, if not controlled by the endogenous antioxidant systems, could lead to a state of oxidative stress. The evidence for accumulation of ROS leading to β-cell dysfunction and apoptosis has been reviewed recently [24,25]. Notably, compared with other mammalian cell types, pancreatic β-cell is very vulnerable to ROS because of weak expression of antioxidant enzymes [26,27]. AGEs are thought to play a major role, at least in significant part by increasing intracellular ROS production, in β-cell damage [7,9,10]. Furthermore, our previous studies [10] and other report [11] have demonstrated that antioxidant therapy protects against AGEs-induced β-cell apoptosis and insulin secretion defect. In the present study, we also observed increased ROS generation in AGE-treated mice islets and MIN6 cells, while sesamin therapy markedly relieved it. Taken together, the effect of sesamin on improving AGEs-induced β-cell damage is attributed, at least in part, to a reduction of oxidative stress.

In pancreatic β-cell, ROS production via non-mitochondrial and mitochondrial pathways has been reported [28]. Both the mitochondrial electron transport chain and NADPH oxidase (an important non-mitochondrial ROS source) pathways participate in AGE-stimulated β-cells [29]. However, the mitochondrial source of ROS occurred in the early period of exposure (in the first 12 h) and NADPH oxidase is the most important source after the long periods of exposure to AGEs (more than 24 h) [10,11]. Indeed, the elevations of p22^phox^ and p67^phox^ expression as well as NADPH oxidase activity were also found in MIN6 cells after stimulated with AGEs for 24 h. Sesamin treatment ameliorated these abnormal changes. These results indicate that sesamin therapy reduces AGE-evoked pancreatic β-cell oxidative stress through an inhibition of NADPH oxidase.

The antioxidant effects of sesamin may be either due to the increase of superoxide dismutase (SOD) activity [30,31] or due to the enhancement of antioxidant activity of vitamin E [32]. Lei *et al.* have reported the protective effects of sesamin on NIT-1 pancreatic β-cell damaged by streptozotocin via the increased activity of SOD [33]. Augmented SOD activity suggests that sesamin could enhance the ability to eliminate superoxide formed during oxidative stress. Furthermore, sesamin has been demonstrated to decrease the expression and activity of NADPH oxidase in the present study. Hsieh *et al.* have shown that treatment with sesamin (30 mg/kg) for 3 days significantly increases 50% of plasma α-tocopherol levels from male FVB mice [34]. Consumption of sesame seed powder over 5 weeks also enhances plasma α-tocopherol levels in healthy human volunteers [35]. Recent studies have suggested that α-tocopherol has a suppressive effect on NADPH oxidase expression and activity *in vitro* [36,37] and *in vivo* [38]. Therefore, the effects of sesamin on reducing of NADPH oxidase may be attributed to the elevated plasma α-tocopherol level and the enhancement of antioxidant effects of α-tocopherol. The molecular mechanisms of sesamin on NADPH oxidase warrants further study.

## 5. Conclusions

The present study demonstrates for the first time that treatment with sesamin improves the AGE-induced β-cell dysfunction and apoptosis both *in vitro* and *in vivo*, and these effects seem to be associated with the attenuation of ROS production mediated by NADPH oxidase inhibition. These findings support that sesamin may be useful in the treatment of DM by protecting against pancreatic β-cell damage.

## References

[1] Peyroux J., Sternberg M. (2006). Advanced glycation endproducts (AGEs): Pharmacological inhibition in diabetes. Pathol. Biol..

[2] Vlassara H., Palace M.R. (2002). Diabetes and advanced glycation endproducts. J. Intern. Med..

[3] Kong X., Ma M.Z., Huang K., Qin L., Zhang H.M., Yang Z., Li X.Y., Su Q. (2014). Increased plasma levels of the methylglyoxal in patients with newly diagnosed type 2 diabetes 2. J. Diabetes.

[4] Wendt T.M., Tanji N., Guo J., Kislinger T.R., Qu W., Lu Y., Bucciarelli L.G., Rong L.L., Moser B., Markowitz G.S. (2003). RAGE drives the development of glomerulosclerosis and implicates podocyte activation in the pathogenesis of diabetic nephropathy. Am. J. Pathol..

[5] Ahmed N., Thornalley P.J. (2007). Advanced glycation endproducts: What is their relevance to diabetic complications?. Diabetes Obes. Metab..

[6] Zhao Z., Zhao C., Zhang X.H., Zheng F., Cai W., Vlassara H., Ma Z.A. (2009). Advanced glycation end products inhibit glucose-stimulated insulin secretion through nitric oxide-dependent inhibition of cytochrome c oxidase and adenosine triphosphate synthesis. Endocrinology.

[7] Coughlan M.T., Yap F.Y., Tong D.C., Andrikopoulos S., Gasser A., Thallas-Bonke V., Webster D.E., Miyazaki J., Kay T.W., Slattery R.M. (2011). Advanced glycation end products are direct modulators of beta-cell function. Diabetes.

[8] Cheng A.S., Cheng Y.H., Lee C.Y., Chung C.Y., Chang W.C. (2015). Resveratrol protects against methylglyoxal-induced hyperglycemia and pancreatic damage *in vivo*. Nutrients.

[9] Ge Q.M., Dong Y., Su Q. (2010). Effects of glucose and advanced glycation end products on oxidative stress in MIN6 cells. Cell. Mol. Biol..

[10] Lin N., Zhang H., Su Q. (2012). Advanced glycation end-products induce injury to pancreatic beta cells through oxidative stress. Diabetes Metab..

[11] Costal F., Oliveira E., Raposo A., Machado-Lima A., Peixoto E., Roma L., Santos L., Lopes F.J., Carpinelli A.R., Giannella-Neto D. (2013). Dual effect of advanced glycation end products in pancreatic islet apoptosis. Diabetes Metab. Res. Rev..

[12] Liu C., Wan X., Ye T., Fang F., Chen X., Chen Y., Dong Y. (2014). Matrix metalloproteinase 2 contributes to pancreatic Beta cell injury induced by oxidative stress. PLoS ONE.

[13] Kong X., Yang J.R., Guo L.Q., Xiong Y., Wu X.Q., Huang K., Zhou Y. (2009). Sesamin improves endothelial dysfunction in renovascular hypertensive rats fed with a high-fat, high-sucrose diet. Eur. J. Pharmacol..

[14] Kong X., Zhang D.Y., Wu H.B., Li F.X. (2011). Losartan and pioglitazone ameliorate nephropathy in experimental metabolic syndrome rats. Biol. Pharm. Bull..

[15] Lee W.J., Ou H.C., Wu C.M., Lee I.T., Lin S.Y., Lin L.Y., Tsai K.L., Lee S.D., Sheu W.H. (2009). Sesamin mitigates inflammation and oxidative stress in endothelial cells exposed to oxidized low-density lipoprotein. J. Agric. Food Chem..

[16] Kiso Y. (2004). 15 Antioxidative roles of sesamin, a functional lignan in sesame seed, and its effect on lipid- and alcohol-metabolism in the liver: A DNA microarray study. Biofactors.

[17] Zhang J.X., Yang J.R., Chen G.X., Tang L.J., Li W.X., Yang H., Kong X. (2013). Sesamin ameliorates arterial dysfunction in spontaneously hypertensive rats via downregulation of NADPH oxidase subunits and upregulation of eNOS expression. Acta Pharmacol. Sin..

[18] Miyawaki T., Aono H., Toyoda-Ono Y., Maeda H., Kiso Y., Moriyama K. (2009). Antihypertensive effects of sesamin in humans. J. Nutr. Sci. Vitaminol..

[19] Hong L., Yi W., Liangliang C., Juncheng H., Qin W., Xiaoxiang Z. (2013). Hypoglycaemic and hypolipidaemic activities of sesamin from sesame meal and its ability to ameliorate insulin resistance in KK-Ay mice. J. Sci. Food Agric..

[20] Makita Z., Vlassara H., Cerami A., Bucala R. (1992). Immunochemical detection of advanced glycosylation end products *in vivo*. J Biol Chem..

[21] Kong X., Zhang Y., Wu H.B., Li F.X., Zhang D.Y., Su Q. (2012). Combination therapy with losartan and pioglitazone additively reduces renal oxidative and nitrative stress induced by chronic high fat, sucrose, and sodium intake. Oxid. Med. Cell. Longev..

[22] Kong X., Ma M.Z., Zhang Y., Weng M.Z., Gong W., Guo L.Q., Zhang J.X., Wang G.D., Su Q., Quan Z.W. (2014). Differentiation therapy: Sesamin as an effective agent in targeting cancer stem-like side population cells of human gallbladder carcinoma. BMC Complement. Altern. Med..

[23] Mizutari K., Ono T., Ikeda K., Kayashima K., Horiuchi S. (1997). Photo-enhanced modification of human skin elastin in actinic elastosis by *N*(epsilon)-(carboxymethyl)lysine, one of the glycoxidation products of the Maillard reaction. J. Investig. Dermatol..

[24] Robertson R., Zhou H., Zhang T., Harmon J.S. (2007). Chronic oxidative stress as a mechanism for glucose toxicity of the beta cell in type 2 diabetes. Cell Biochem. Biophys..

[25] Lupi R., Del P.S. (2008). Beta-cell apoptosis in type 2 diabetes: Quantitative and functional consequences. Diabetes Metab..

[26] Lenzen S., Drinkgern J., Tiedge M. (1996). Low antioxidant enzyme gene expression in pancreatic islets compared with various other mouse tissues. Free Radic. Biol. Med..

[27] Lenzen S. (2008). Oxidative stress: The vulnerable beta-cell. Biochem. Soc. Trans..

[28] Turrens J.F. (2003). Mitochondrial formation of reactive oxygen species. J. Physiol..

[29] Guichard C., Moreau R., Pessayre D., Epperson T.K., Krause K.H. (2008). NOX family NADPH oxidases in liver and in pancreatic islets: A role in the metabolic syndrome and diabetes?. Biochem. Soc. Trans..

[30] Su S., Li Q., Liu Y., Xiong C., Li J., Zhang R., Niu Y., Zhao L., Wang Y., Guo H. (2014). Sesamin ameliorates doxorubicin-induced cardiotoxicity: Involvement of Sirt1 and Mn-SOD pathway. Toxicol. Lett..

[31] Lahaie-Collins V., Bournival J., Plouffe M., Carange J., Martinoli M.G. (2008). Sesamin modulates tyrosine hydroxylase, superoxide dismutase, catalase, inducible NO synthase andinterleukin-6 expression in dopaminergic cells under MPP+-induced oxidative stress. Oxid. Med. Cell. Longev..

[32] Ghafoorunissa S., Hemalatha S., Rao M.V. (2004). Sesame lignans enhance antioxidant activity of vitamin E in lipid peroxidation systems. Mol. Cell. Biochem..

[33] Lei H., Han J., Wang Q., Guo S., Sun H., Zhang X. (2012). Effects of sesamin on streptozotocin (STZ)-induced NIT-1 pancreatic β-cell damage. Int. J. Mol. Sci..

[34] Hsieh P.F., Hou C.W., Yao P.W., Wu S.P., Peng Y.F., Shen M.L., Lin C.H., Chao Y.Y., Chang M.H., Jeng K.C. (2011). Sesamin ameliorates oxidative stress and mortality in kainic acid-induced status epilepticus by inhibition of MAPK and COX-2 activation. J. Neuroinflammation.

[35] Wu W.H., Kang Y.P., Wang N.H., Jou H.J., Wang T.A. (2006). Sesame ingestion affects sex hormones, antioxidant status, and blood lipids in postmenopausal women. J. Nutr..

[36] Palipoch S., Punsawad C., Koomhin P., Suwannalert P. (2014). Hepatoprotective effect of curcumin and alpha-tocopherol against cisplatin-induced oxidative stress. BMC Complement. Altern. Med..

[37] Thakurta I.G., Chattopadhyay M., Ghosh A., Chakrabarti S. (2012). Dietary supplementation with N-acetyl cysteine, α-tocopherol and α-lipoic acid reduces the extent of oxidative stress and proinflammatory state in aged rat brain. Biogerontology.

[38] Pazdro R., Burgess J.R. (2012). Differential effects of α-tocopherol and N-acetyl-cysteine on advanced glycation end product-induced oxidative damage and neurite degeneration in SH-SY5Y cells. Biochim. Biophys. Acta.

